# Concise Review: Evidence for CD34 as a Common Marker for Diverse Progenitors

**DOI:** 10.1002/stem.1661

**Published:** 2014-05-23

**Authors:** Laura E Sidney, Matthew J Branch, Siobhán E Dunphy, Harminder S Dua, Andrew Hopkinson

**Affiliations:** a Academic Ophthalmology, Division of Clinical Neuroscience, University of Nottingham, Queen's Medical Centre CampusNottingham, United Kingdom; b Tissue Engineering and Drug Delivery, School of Pharmacy, University of NottinghamNottingham, United Kingdom

**Keywords:** CD34, Stem cell, Progenitor, Mesenchymal, Stromal, Epithelial, Endothelial

## Abstract

CD34 is a transmembrane phosphoglycoprotein, first identified on hematopoietic stem and progenitor cells. Clinically, it is associated with the selection and enrichment of hematopoietic stem cells for bone marrow transplants. Due to these historical and clinical associations, CD34 expression is almost ubiquitously related to hematopoietic cells, and it is a common misconception that CD34-positive (CD34^+^) cells in nonhematopoietic samples represent hematopoietic contamination. The prevailing school of thought states that multipotent mesenchymal stromal cells (MSC) do not express CD34. However, strong evidence demonstrates CD34 is expressed not only by MSC but by a multitude of other nonhematopoietic cell types including muscle satellite cells, corneal keratocytes, interstitial cells, epithelial progenitors, and vascular endothelial progenitors. In many cases, the CD34^+^ cells represent a small proportion of the total cell population and also indicate a distinct subset of cells with enhanced progenitor activity. Herein, we explore common traits between cells that express CD34, including associated markers, morphology and differentiation potential. We endeavor to highlight key similarities between CD34^+^ cells, with a focus on progenitor activity. A common function of CD34 has yet to be elucidated, but by analyzing and understanding links between CD34^+^ cells, we hope to be able to offer an insight into the overlapping properties of cells that express CD34. Stem Cells *2014;32:1380–1389*

## Introduction

CD34 is predominantly regarded as a marker of hematopoietic stem cells (HSC) and hematopoietic progenitor cells. However, CD34 is now also established as a marker of several other nonhematopoietic cell types, including vascular endothelial progenitors 1 and embryonic fibroblasts 2. Accumulating evidence demonstrates CD34 expression on several other cell types, including multipotent mesenchymal stromal cells (MSC), interstitial dendritic cells, and epithelial progenitors 3–6, but there remains limited recognition of the role of CD34-positive (CD34^+^) cells outside of each individual specialty. Despite consistent evidence of expression by many cell types, there is still a misconception that CD34 represents a cell of hematopoietic origin, and experimentally, CD34^+^ cells are often regarded as hematopoietic contamination and subsequently disregarded.

This review presents evidence establishing CD34 as a general marker of progenitor cells. We explore common traits, such as marker expression, morphology and differentiation potential, and endeavor to draw focus toward the many, disparate cell types that express CD34, and in the process highlight key similarities. CD34 expression across different cell types and the associated implications has not previously been presented, although selected literature has reviewed expression within individual cell groups. Although a common function of CD34 has yet to be elucidated, analyzing and understanding the links between cells offers an insight into the role of CD34 in identifying progenitor cells from many tissue types. A summary of the properties of all the CD34^+^ cell types discussed in this review can be found in Table1.

**Table 1:** Summary of different CD34^+^ cell types

CD34^+^ cell type	Associated markers	Differentiation potential	Properties	Reference
HSC and progenitors	HLA-DR, CD38, CD117 (c-kit), CD45, CD133	Hematopoietic cells, cardiomyocytes, hepatocytes	Large nucleus, little cytoplasm, high proliferative capacity	7, 8
MSC	Stro-1, CD73, CD90, CD105, CD146, CD29, CD44, CD271	Adipogenic, osteogenic, chondrogenic, myogenic, angiogenic	CD34^+^ MSC form a higher proportion of CFU-f colonies than CD34^−^. CD34^+^ MSC exhibit a high proliferative capacity. Fibroblastic cells	9–13
Muscle satellite cells	CD56, Myf5, Desmin, M-cadherin, CD90, CD106, Flk-1, VEGFR, MyoD, CD146	Myogenic, adipogenic, osteogenic, chondrogenic	The CD56^+^CD34^+^ population may represent a more primitive or pluripotent stem cell. In vivo, CD34^+^ cells are located near the basal lamina. Small and round	14–17
Keratocytes	CD34, CD133, l-selectin, keratocan, ALDH	Fibroblastic, myofibroblastic, adipogenic, osteogenic, chondrogenic, corneal epithelial, corneal endothelial	Dendritic morphology. In vitro population acquires an MSC phenotype	18–21
Interstitial cells	CD117, vimentin, Desmin, Connexin-43, PDGFRβ	Not yet fully elucidated	Triangular or spindle-shaped with large nucleus and long cytoplasmic processes. CD34^+^ population may have a stem cell/progenitor role in the bladder, intestine, and reproductive organs	22–24
Fibrocyte	CD45, CD80, CD86, MHC class I and II	Fibroblastic, myofibroblastic, adipogenic, osteogenic, chondrogenic	Small spindle shape. CD34 is lost in culture and upon maturation	25–27
Epithelial progenitors	CD49f, CD10, CD146, CD71, S100a4, Dkk3, CD133, CD117, ALDH, CD90	Dermal epithelial cells, neural mesenchymal	Predominantly described in HF niche in skin	28–33
Endothelial cells	CD146, VE-cadherin, CD133, CD117, CD14, CD31	Angiogenesis	Elongated with filopodia. Lack tight junctions. CD34 is present on luminal membrane processes and is expressed on filopodia during in vivo angiogenesis. Quiescent in vivo/low proliferation activity	1, 34, 35

Abbreviations: ALDH, aldehyde dehydrogenase; CD, cluster of differentiation; CFU-F, colony forming units fibroblast; Flk-1, fetal liver kinase-1; HF, hair follicle; HLA-DR, human leukocyte antigen-DR; HSC, hematopoietic stem cells; MSC, multipotent mesenchymal stromal cells; Myf5, myogenic factor 5; MyoD, myogenic differentiation 1; MHC, major histocompatibility complex; PDGFRβ, platelet derived growth factor receptor β; VEGFR, vascular endothelial growth factor receptor.

## Structure and Function of CD34

CD34 is a transmembrane phosphoglycoprotein, first identified in 1984 on hematopoietic stem and progenitor cells 36. It has a molecular weight of approximately 115 kDa and possesses an extracellular domain that is heavily sialylated, O-linked glycosylated, and contains some N-linked glycosylation sites. There is a single transmembrane helix and a cytoplasmic tail that contains PDZ (PSD-95-Dlg-ZO-1)-domain binding motifs 3,37. The most commonly described ligand for CD34 is l-Selectin (CD62L), however, the adapter protein CrkL, known for adhesion regulation, also binds CD34 38,39.

Although the structure of CD34 is well-investigated, there is still relatively little known about its function. Studies in hematopoietic cells suggest roles in cytoadhesion and regulation of cell differentiation and proliferation 40,41. Lymphocytes exhibit l-selectin-mediated adhesion to CD34 surface proteins in the vascular endothelium 38,42 and in addition, it has been hypothesized that CD34 plays a role in trafficking of HSC to niches within the bone marrow (BM) 41. However in contrast, CD34 has also been associated with blocking of adhesion, particularly involving mast cells 43.

### CD34 and Hematopoietic Cells

The expression of CD34 on hematopoietic progenitors and the properties of these cells have been discussed in depth previously 7,44,45 and are not covered in detail in this review. In clinical practice, CD34 expression is evaluated to ensure rapid engraftment in BM transplants and can also be used as a selective marker in cell sorting to enrich a population of immature hematopoietic cells 46,47. Although sometimes assumed to be solely a stem cell marker, the detection of CD34 in BM or blood samples represents a hematopoietic stem/progenitor mix, of which the majority of cells are progenitor 44. Human HSC are further separated from CD34^+^ progenitor cells by low expression of CD90 and a lack of expression of CD38, human leukocyte antigen-DR, and a panel of mature hematopoietic lineage markers (lin^−^) 7. CD34^+^ HSC are able to differentiate into all cells of the hematopoietic lineage and have a high proliferative capacity 7,8. Evidence suggests that CD34^+^ HSC and progenitors have the ability to differentiate in vivo into other lineages, including respiratory epithelial cells 48, hepatocytes 49, and cardiomyocytes 50. Thus far, the properties of CD34^+^ HSC have not been directly linked to the properties of CD34^+^ non-HSC.

## CD34 and Stromal Cells

### Multipotent MSC

MSC are found in most adult tissues and are a prevalent and versatile cell type, studied extensively for regenerative medicine applications 51. Although their in vitro mesenchymal differentiation potential is well-reported, MSC are also frequently associated with other properties including paracrine wound healing, niche forming abilities, immune privilege, and immunomodulation 52,53. Nomenclature of MSC is a controversial topic, with differing opinions on whether cells should be identified as mesenchymal stem cells or multipotent mesenchymal stromal cells, among many other names 54. In this review, we use the term multipotent mesenchymal stromal cells but include a broad range of references that refer generically to mesenchymal stem and progenitor cells. This section includes research on MSC from different tissue sources and discusses both freshly isolated and culture expanded MSC.

Two recent reviews have discussed the expression of CD34 on MSC, both with an emphasis on adipose-derived MSC 4,55. While Scherberich et al. 55 concentrated on the structure and function of CD34 in relation to MSC, Lin et al. 4 challenged the opinion that CD34 is a negative marker of MSC. The latter review discussed evidence demonstrating that CD34 was an important marker in early MSC research, highlighting research by Simmons and Torok-Storb showing that freshly isolated, CD34^+^ BM MSC form greater proportions of fibroblastic colonies (colony forming units fibroblast) than their CD34^−^ counterparts 9. The review also made the observation that CD34^+^ MSC were originally used in screening for additional MSC immunogens, leading to the identification of Stro-1 56. They concluded that CD34 should be considered a positive marker of MSC, with a particular association to vasculature and suggested that these cells be known as vascular stem cells 4. While Lin et al. highlights several key issues in the definition of an MSC, the review only briefly touches upon a connection between CD34 and progenitor cells.

This same review was also critical of the position paper, published in 2006, by the International Society for Cellular Therapy (ISCT) 57, which outlined a minimal criteria for cells to be considered MSC. These criteria, based predominantly on MSC extracted from BM, describe a plastic-adherent, culture expanded cell population and have subsequently been widely adopted by the research community. One ISCT criterion states that to be considered MSC, a cell population must be largely negative for CD34 (≤2% of the population). This is due to the consideration that CD34 is a marker of hematopoietic cells, alongside a number of previous studies that show an absence of CD34 in cultured MSC 58–60. In support of the ISCT, establishing well-defined criteria has created a clear benchmark for the depiction of MSC populations, allowing more accurate and reproducible comparisons between research groups. MSC populations demonstrate high levels of heterogeneity and this diversity exists not only within different tissue sources but also between MSC from a single source and clonal MSC 60,61. Hence, prior to the establishment of these criteria, many disparate cell types were described as MSC, leading to uncertainty over MSC properties and characteristics. However, in unifying and defining characterization, it is almost certain that stromal progenitors expressing markers that are considered negative, or not included in the ISCT criteria, are being overlooked by researchers, and more importantly, actively removed when following these guidelines.

Although prevailing opinion states that MSC are CD34^−^, in vitro characterization predominantly takes place following culture, usually after several passages. Consequently, this is not representative of the in vivo or initially extracted cell phenotype. Freshly extracted stromal cells, from various tissues, have been shown to contain CD34^+^ cells 9–11,63. The expression of CD34 has been demonstrated more extensively on MSC isolated from sources other than BM, such as adipose tissue, and therefore is more widely accepted by researchers in these areas 10,61,64. CD34^+^ MSC are present immediately following extraction but rapidly diminish in numbers after a short time in culture 12,13,65. For example, when analyzing adipose-derived MSC at P0, Mitchell et al. described an average of 59.2% of adherent cells expressing CD34, which decreased to 5% at passage 2 and subsequently disappeared 64. This change in CD34 expression upon culture is also mirrored in changes of expression of other surface antigens. Freshly extracted adipose MSC also show low expression of CD73, CD90, and CD105 that increase upon culture; this is of interest due to the ISCT classification of these as definitive MSC markers 10,64. There are also decreases in expression of other markers associated with MSC, such as CD106, CD146, and CD271 12,13,64,66. This change in MSC phenotype upon culture is perhaps indicative of a differentiation process or response to environmental changes.

CD34^+^ cells represent a proportion of the total MSC population, and this subset of cells possesses distinct characteristics. CD34 expression is associated with high colony forming efficiency and long-term proliferative capacity 9,12,63. CD34^+^ MSC have been associated with typical MSC markers, alongside differentially expressed markers such as CD271 and Stro-1, and markers commonly related to other cell types including CD45 and CD133 10,12,13,41,42,67,68. CD34^+^ MSC have been shown to possess a greater propensity for endothelial transdifferentiation 14,69. CD34 is also found on embryonic stem cell derived MSC, further suggesting it to be a marker of early human MSC 15.

### Muscle Satellite Cells

CD34 is commonly used as a marker of both human and mouse muscle satellite cells, also known as muscle stem cells 16,70. Muscle satellite cells are small, stromal progenitor cells that give rise to mature skeletal muscle cells. In vivo, muscle satellite cells are quiescent, unless activated to provide myonuclei for myofibers, by increased weight-bearing exercise or trauma. Activation of these cells, also considered to be differentiation, corresponds to a complete downregulation of CD34 71. CD34 has been suggested to play a fundamental role in the regulation of muscle progenitor cell differentiation and establishment and maintenance of a satellite cell population 71.

CD34 is not expressed on all muscle satellite cells but is used in identification alongside other markers including CD56 16,17. Developmentally, the earliest known myogenic precursors do not express CD34 and during muscle development CD34 expression is first detected with the appearance of committed muscle satellite cells 72. This, alongside the simultaneous expression of myogenic markers such as Myf5 and M-cadherin, is suggestive of CD34^+^ satellite cells demonstrating a prior commitment to the myogenic fate 71.

Alternative evidence proposes that CD34^+^ cells have the potential to be more than myogenic precursors. Distinct MSC-like cells, that demonstrate in vitro mesenchymal differentiation, have been identified in muscle satellite locations by characterizing CD34 expression 73. Similar to MSC, CD34 expression on muscle satellite cells does not persist during culture and disappears as the cells differentiate. It has been suggested that CD34^+^ cells residing in the interstitial spaces of muscle are related to endothelial cells, due to the expression profile CD56^+^CD34^+^CD144^+^ 18. These myoendothelial cells show an enhanced ability to regenerate muscle, compared to cells that express only myogenic or endothelial markers in an in vivo mouse model. They also differentiate in vitro down the osteogenic and chondrogenic lineages.

The different observed properties of muscle satellite cells may be explained by the presence of distinct subsets of CD34^+^ cells, with distinct differentiation potentials 17. The markers that are coexpressed alongside CD34 have an impact on differentiation. For example, CD34^+^ cells that coexpress the endothelial marker CD31 display angiogenic differentiation. However, CD34^+^CD31^−^ cell populations demonstrate greater potential to differentiate down the adipogenic and myogenic lineage 17.

### Corneal Keratocytes

**Figure 1:**
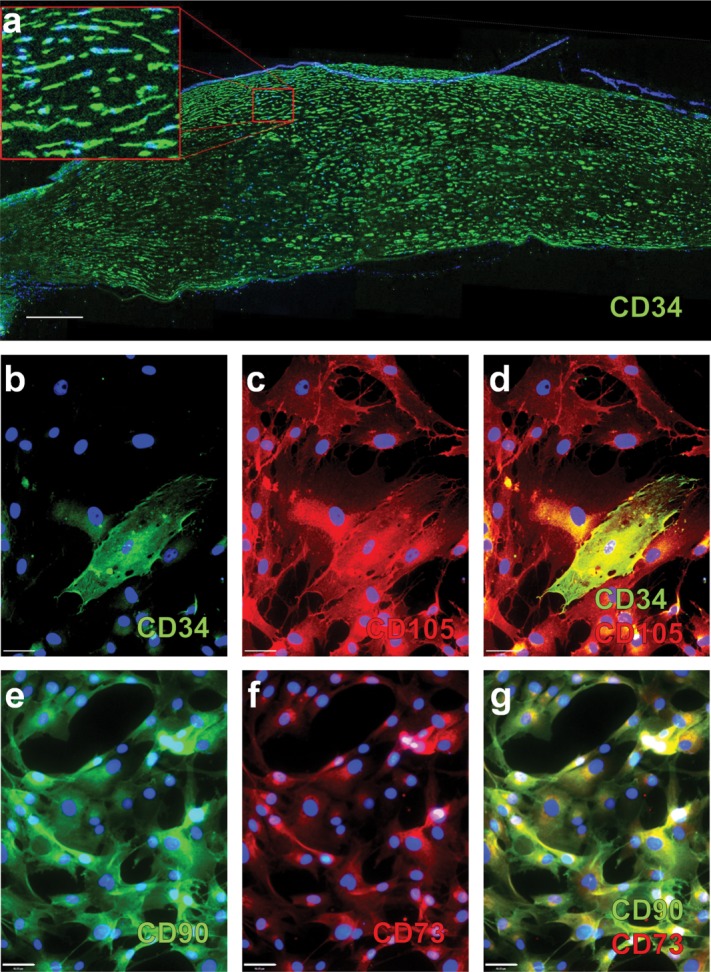
CD34 expression by keratocytes. (A): Immunofluorescence showing CD34 expression by keratocytes in a section of human cornea, counterstained with DAPI. Scale bar = 500 μm. (B–G): Keratocyte phenotype after in vitro culture. Human keratocytes were extracted from the corneal stroma and cultured for 5 days in Medium 199 containing 20% fetal bovine serum. (B–D): Immunocytochemistry identifying individual cells expressing CD34, among cells expressing CD105. (E–G): Keratocytes in culture also express markers associated with MSC, such as CD90 and CD73. All images counterstained with Hoechst 33258. Scale bar = 46 μm.

The corneal stroma is populated with quiescent cells known as keratocytes, which exhibit a dendritic morphology with extensive cellular contacts 19. Similar to muscle satellite cells, CD34 is a well-established marker for quiescent keratocytes in vivo (Fig. 1) 74,75. As a result of corneal trauma, keratocytes adjacent to the wound take on a more fibroblastic phenotype and CD34 expression rapidly disappears, as cells become “activated” 20. Upon activation, they shift to a fibroblastic phenotype and are associated with stromal tissue remodeling and scar formation 76. Activation also occurs in vitro, when keratocytes are cultured on tissue culture plastic, particularly in serum-containing medium 65,77. Keratocyte transformation to the fibroblast phenotype was previously thought to be irreversible in vitro, but recent evidence demonstrates that careful recapitulation of the niche-like environment and tailoring of culture conditions has the potential to revert fibroblastic cells back to a native keratocyte phenotype 78–80. However, it has yet to be investigated whether CD34 expression is restored.

The function of CD34 expression in keratocytes has not yet been elucidated, although it has been speculated that CD34 plays roles in regulation of differentiation, adhesion, and quiescence 75. Although it has been suggested that CD34^+^ keratocytes are of hematopoietic origin 81, these cells do not form hematopoietic colonies when cultured in semisolid medium and CD34^+^ cells from these cultures display a plastic adherent, dendritic morphology 65. Although CD34^+^ cells disappear during in vitro culture, they can still be seen in early cultures, when cultured in medium 199 (Fig. 1B). These cells also express typical stromal markers such as CD105, CD90, and CD73 (Fig. 1C–1G). In vitro, keratocytes have been shown to display characteristics of MSC 21,82, and after several passages, when CD34 has disappeared, conform to the requisite ISCT criteria 65. Therefore, it has been hypothesized that the keratocyte is an MSC progenitor found in the corneal stroma 65,83. Additionally, recent investigations have revealed that CD34^+^ keratocytes possess the ability to transdifferentiate into corneal epithelial 77 and endothelial cells 22,74. This has led to the hypothesis that CD34 is a marker for a tissue-specific progenitor that resides in the stroma, which could give rise to all corneal lineages and MSC.

### Interstitial Cells and Fibrocytes

CD34^+^ stromal cells have been described in organs including the thyroid, dermis, tonsils, uterus, and testes 84. CD34^+^ cells in these cases are termed dendritic interstitial cells or fibrocytes. Although their function remains unclear, these cells display similar properties to CD34^+^ MSC, keratocytes, and muscle satellite cells, and it has been suggested that these cells function as progenitor cells for specific tissues.

The CD34^+^ interstitial cells of Cajal (ICC) are mesenchymal in origin and were first identified in the gastrointestinal (GI) tract 85. The prevailing belief is that ICC are responsible for modulating smooth muscle contraction through electrical and chemical signaling as pacemaker cells. However, the discovery of ICC in other tissues, including the pancreas, myocardium, bladder, urethra, and blood vessels, has brought into question the functional role of these cells 86.

Although not all ICC express CD34, immunoreactivity has been shown in a subset of ICC in the GI tract in mice and humans 86. In humans, CD34^+^ populations have been described in the human detrusor muscle of the bladder, uterus, and fallopian tubes 23,24. Investigations have identified a possible role for CD34^+^ ICC as uncommitted progenitor cells 25. Moreover, ICC in the bladder of mice showed colocalization of c-kit and CD34 26 and authors have suggested a functional relationship between CD34^+^ ICC and mast cells. This supports the notion that CD34 expression relates to plasticity of the ICC cell type and a progenitor function.

Fibrocytes are mesenchymal progenitor cells that are often considered distinct from MSC. Despite this, they are quiescent cells that circulate in the bloodstream and upon trauma are recruited to the site of injury, where the cells play roles in inflammation and wound healing 27,87. These cells have been shown to produce myofibroblastic, fibroblastic, adipogenic, chondrogenic, and osteogenic phenotypes in vitro 27,28. Fibrocytes are currently not recognized as MSC due to their cell surface marker profile which includes markers associated with leukocytes, including CD34, CD45, CD80, CD86, and major histocompatibility complex class I and II 27,28. As with the aforementioned stromal cells, CD34 expression disappears during in vitro culture and in vivo upon maturity and differentiation 87. In common with other stromal cells, it has been suggested that both keratocytes and fibrocytes are derived from leukocytes due to a CD34^+^CD45^+^ cell surface marker profile 81.

## CD34 and Epithelial Cells

The expression of CD34 by epithelial progenitors in the skin is widely accepted 29,88. In vivo, each unit of epithelium contains a hair follicle (HF) and within the HF there is a stem cell niche that maintains the epithelial layers of the skin. Trauma to the epidermis results in migration of stem cells from this niche to the injury site. Within the niche there are populations of multipotent epithelial stem cells and a subpopulation of these cells is CD34^+^ 29,89. The location of the CD34^+^ cells in the HF is one of contention, depending on species. The majority of studies have been conducted on mice and in these cases the CD34^+^ population resides in the outer root sheath (ORS) bulge, alongside other recognized stem cells 6,29. Human HFs are considerably more difficult to study, due to small size and low cell numbers. However, indications show that the CD34^+^ cells are not located in the ORS bulge, but are below the bulge zone, in the suprabulbar region 89–91. In humans, CD34^+^ cells have also been identified in the skin between the HF, in the basal interfollicular epidermis, but these cells are less well-investigated 92. It has been suggested that because the CD34^+^ cells are not present in the adult human ORS bulge, with the majority of other stem cells, they are the progeny of bulge stem cells 92.

HFs are constantly cycling through stages of active hair growth (anagen), destruction (catagen), and rest (telogen). During these stages, cell surface marker expression and phenotype within the HF is altered 6. CD34 expression is seen in human HFs during anagen but not during catagen and telogen 89. The authors suggest that this indicates that CD34 function relates only to epithelial cells that are proliferating and also relates to adhesion of the root sheath cells to the surrounding stroma. It is worth noting that although human CD34^+^ cells are not located in the bulge with other stem cells, they are located in the area of the HF that demonstrates the most clonogenic activity 30. In human HFs, cells that express CD34 do not express cytokeratin 15 (CK15), another commonly used marker of epithelial progenitor cells 31, suggesting either more than one population of progenitor cells or a hierarchy of cells at different stages of differentiation 89. CD34^+^ cells of the HF are multipotent and able to generate a fully stratified epidermis 6,32. There is some evidence to show that HF stem cells, including CD34^+^ cells, may be pluripotent, demonstrating transdifferentiation into neural and mesenchymal lineages 33,93.

The majority of research into CD34 expression in epithelial cells has been performed on skin tissue. However, there is a population of CD34^+^ stem cells residing in the epithelial ducts of the salivary gland 94,95. These cells retain their proliferative ability when cultured in 3D structures known as salispheres and demonstrate differentiation into cells and structures reminiscent of the salivary gland. There are also some links between CD34^+^ stromal cells and epithelial cells. CD34^+^ keratocytes have been shown to express the epithelial cell marker CK3 96, and CD34^+^ cells from human fetal liver express biliary epithelial markers CK7, CK8, and CK18 97.

## CD34 and Endothelial Cells

CD34 is widely regarded as a marker of vascular endothelial progenitor cells 1,98. These BM-derived cells are found circulating in peripheral blood 99 and their usefulness in proangiogenic therapies has been extensively researched 98,99. The properties of CD34^+^ endothelial cells are often linked with hematopoietic cells, as both cell types can be isolated from peripheral blood using CD34 as an antigen. CD34^+^ cells isolated from peripheral blood have been studied for use in neovascularization therapies 100 and furthermore have shown ability to differentiate into cardiomyocytes 50 and osteoblasts 34.

A review published several years ago by Matsumoto et al. 35 discussed a possible overlap between endothelial progenitor cells and osteoblasts. There is a circulating CD34^+^ population of endothelial/skeletal progenitor cells, thought to originate in BM, capable of differentiating into both osteoblasts and endothelial cells. Investigations are ongoing using the circulating CD34^+^ cells to treat cases of nonunion fractures, which often suffer from delayed healing times due to inadequate blood supply around the injury site.

There is a subset of noncirculating adult endothelial cells that are also CD34^+^, most notably located within smaller blood vessels, while most endothelial cells in larger veins and arteries are CD34^−^ 1. In contrast to the typical cobblestone morphology of endothelial cells, CD34^+^ cells are more elongated and lack tight junctions 101. CD34 expression is predominantly found on the luminal membrane of cellular processes but may also be seen on the abluminal membrane of cells found at the tips of vascular sprouts 1,102. In addition, CD34^+^ endothelial cells are quiescent and are thought to be involved in migration and adhesion 1.

Human umbilical vein endothelial cells (HUVEC) are CD34^+^ in vivo, however, when cultured in vitro, expression is lost after several passages and only a small population of CD34^+^ cells are retained 1. CD34^+^ HUVEC have distinct morphological characteristics, including numerous filopodia 101. Angiogenic stimuli provokes migration of these cells, and it has been proposed that this CD34^+^ subpopulation is homologous to sprouting tip cells, a specialized type of endothelial cell present at the leading edge during in vivo angiogenesis 101. CD34 is strongly expressed on the filopodia of these tip cells at sites of active angiogenesis and evidence yet again emphasizes the important functional role for CD34 in progenitor cell activity.

## CD34 Antibody Selection

Careful selection of the antibody used for CD34 sorting and identification is of utmost importance during investigations. Some CD34 monoclonal antibodies (mAbs) select for epitopes that are sensitive to cleavage with neuraminidase (sialidase) and glycoprotease 103. Therefore, they are dependent on sialic acid residues remaining on the antigen. For this reason, an epitope classification for CD34 mAbs was devised, as seen in Table2. There are over 30 different mAbs for CD34 targeting different epitopes, and antibody clone used by studies discussed in this review can be seen in Table2. The most commonly used clones are MY10, a class Ib mAb, that is partially dependent on the presence of sialic acid residues and QBEnd10, a class II mAB, that has been shown to successfully detect both sialylated and desialylated CD34 103. The QBEnd10 clone is the antibody of choice for immunohistochemical protocols as it is resistant to denaturation and as the epitope is at the N-terminus of the CD34 molecule it is ideally suited to selection protocols such as fluorescence-activated cell sorting and magnetic-activated cell sorting. Class III epitopes, such as 8G12 retain high specificity for all glycoforms of the CD34 antigen and so are suitable for many protocols including flow cytometry and immunofluorescence 103. Polyclonal antibodies should be avoided for CD34 sorting and immunoblotting due to the possibilities of nonspecific labeling and high variation between antibody batches.

**Table 2:** CD34 antibody selection: epitope specificity and clones

Epitope class	Clone	Cell type	Assays performed	Reference
Ia. Sensitive to neuraminidase and glycoprotease	12.8	Hematopoietic cells, MSC, endothelial progenitors	Immunoblots, immunostaining, FACS	19, 47
	BI.3C5	MSC, keratocytes, endothelial progenitors	Immunoblots, immunostaining, FACS	1, 9, 75
Ib. Partially sensitive to neuraminidase and glycoprotease	ICH3	MSC, keratocytes, endothelial progenitors	Immunoblots, immunostaining, FACS	1, 9, 75, 102
	MY10	Hematopoietic cells, MSC, interstitial cells, endothelial progenitors	Immunoblots, immunostaining, FACS, Western blots	1, 5, 9, 36, 39, 56, 84
II. Resistant to neuraminidase and sensitive to glycoprotease	QBEnd10	MSC, muscle satellite cells, keratocytes, interstitial cells, HF epithelial progenitors, endothelial progenitors.	Immunoblots, immunostaining, flow cytometry	1, 16, 23, 25, 65, 77, 86, 89, 101, 102
III. Resistant to neuraminidase and glycoprotease	563	HF epithelial progenitors	Immunostaining	90
	581	Muscle satellite cells, keratocytes	Immunostaining, flow cytometry	16, 75
	8G12	Muscle satellite cells, haematopoietic cells, MSC	Immunostaining, flow cytometry, FACS	8, 12, 40, 44, 62, 64, 73
	TUK3	Endothelial progenitors	Immunoblots	1
	115.2	Endothelial progenitors	Immunoblots	1
Monoclonal (clone not stated)		MSC	Immunostaining, flow cytometry	10, 58, 59, 63, 69
Polyclonal		HF epithelial	Flow cytometry	91
Antibody type not stated		MSC, muscle satellite cells, keratocytes, HF epithelial progenitors, salivary epithelial progenitors, endothelial progenitors	Immunostaining, flow cytometry, FACS	13–15, 18, 21, 30, 34, 60, 67, 68, 70, 74, 82, 83, 92, 94

Abbreviations: FACS, fluorescence-activated cell sorting; HF, hair follicle; MSC, multipotent mesenchymal stromal cells.

Almost all methods of cell surface marker analysis involve the binding of antibodies to an antigen. Antigen–antibody interactions are noncovalent and can be reversible; therefore, it is important to validate an experiment carefully. Key proteins of interest can be interrogated using multiple methods, such as using an alternative antibody, technique or analysis of gene transcription. To characterize a cell population it is essential to develop a comprehensive characterization process comprising of a profile of markers, both surface and intracellular, functional assays and, as in the case of stem cells, interrogation of their differentiation potentials.

## Conclusion

CD34 is a cell surface marker that is expressed by a broad range of cells including hematopoietic, stromal, epithelial, and endothelial cells. Although the function of CD34 as a surface antigen is still unknown, it has been linked to inhibition or facilitation of adhesion, cell proliferation, and regulation of differentiation 40,41,55,103. This review focused on the use of CD34 to identify cells from diverse tissues that have comparable properties (Table3). The discernible link between all CD34^+^ cell types is progenitor and stem cell activity, and in many cases, the CD34^+^ population of cells shows a more potent or pronounced differentiation capacity. Research alludes to a correlation between cell plasticity and CD34 expression, with loss of CD34, alongside other cell surface antigens, suggesting lineage commitment from the more positive progenitor cell. Many of these cells also demonstrate a quiescent state in vivo, until activated to differentiate. Although all the cell types discussed throughout this review express CD34, they do not all display identical properties. Many coexpress tissue-specific markers alongside CD34, suggesting that the presence of CD34 may indicate a specific progenitor for that tissue. Evidence for this is certainly apparent in muscle satellite cells, keratocytes, and epithelial progenitors. However, this does not necessarily limit the differentiation capacity of the cells in vitro, with many of the CD34^+^ cells linked by transdifferentiation ability.

**Table 3:** Comparable properties of CD34^+^ cells

Comparable properties of CD34^+^ cells	Described in:
Potential stem/progenitor cell population	HSC, MSC, muscle satellite cells, keratocytes, interstitial cells, fibrocytes, HF epithelial stem cells, vascular endothelial progenitors
Quiescent in vivo	Muscle satellite cells, keratocytes, interstitial cells, fibrocytes
Greater CFU-F capacity and high proliferative capacity in vitro	HSC, MSC, HF epithelial stem cells
Loss of CD34 expression upon “activation” or differentiation either in vivo or upon in vitro culture	MSC, muscle satellite cells, keratocytes, fibrocytes, HF epithelial stem cells
Coexpression with tissue specific markers	Muscle satellite cells, keratocytes, interstitial cells, HF epithelial stem cells, salivary gland stem cells, vascular endothelial progenitors
Transdifferentiation potential	HSC, MSC, muscle satellite cells, keratocytes, HF epithelial stem cells, vascular endothelial progenitors

Abbreviations: CD, cluster of differentiation; CFU-F, colony forming units fibroblast; HF, hair follicle; HSC, hematopoietic stem cells; MSC, multipotent mesenchymal stromal cells.

Although CD34 may be one marker that is useful in identifying progenitor populations, a single marker alone is not appropriate for the characterization of a cell type. The expression of CD34 by all cell types discussed throughout the review may not be exclusive. Due to a lack of cohesive research, we cannot yet identify another marker that appears on all cells, but markers such as CD90, CD117 (c-kit), CD146, and CD133 have been indicated on more than one cell type. To fully characterize a population of stem cells it is most likely that a specific marker profile would be required, alongside clonal assays, differentiation assays and functional profiling.

The culture and propagation of adherent CD34^+^ cells in vitro is challenging. This is conceivably due to the association of CD34 with quiescence and adhesion, and the loss of CD34 expression upon differentiation. Culture on tissue culture plastic and in serum-containing medium creates an environment dissimilar to the in vivo environment, forcing the cells to proliferate and differentiate, subsequently losing CD34. If CD34^+^ cells are to be investigated in vitro, more thought, specialized culture conditions and optimization is required to recapitulate an environment more similar to the in vivo niche.

By compiling this review, we would like to propose that CD34 is considered as a surface antigen suitable for the selection of subpopulations of progenitor cells from larger cell populations, including mesenchymal cells, and is not associated only with hematopoietic and endothelial cells. Recognizing CD34 as a progenitor marker will allow further research into this distinct subset of cells, which potentially possess a pronounced differentiation capacity. If culture and propagation techniques for these cells can be optimized, CD34^+^ cells from many tissue types may represent a source of progenitor cells that can be exploited clinically in regenerative medicine strategies.
